# Validation of experimental molecular crystal structures with dispersion-corrected density functional theory calculations

**DOI:** 10.1107/S0108768110031873

**Published:** 2010-09-11

**Authors:** Jacco van de Streek, Marcus A. Neumann

**Affiliations:** a Avant-garde Materials Simulation, Merzhauser Str. 177, D-79100 Freiburg im Breisgau, Germany

**Keywords:** dispersion-corrected density functional theory, organic structures

## Abstract

This paper describes the validation of a dispersion-corrected density functional theory (d-DFT) method for the purpose of assessing the correctness of experimental organic crystal structures and enhancing the information content of purely experimental data. 241 experimental organic crystal structures from the August 2008 issue of *Acta Cryst.* Section E were energy-minimized in full, including unit-cell parameters. The differences between the experimental and the minimized crystal structures were subjected to statistical analysis. The r.m.s. Cartesian displacement excluding H atoms upon energy minimization with flexible unit-cell parameters is selected as a pertinent indicator of the correctness of a crystal structure. All 241 experimental crystal structures are reproduced very well: the average r.m.s. Cartesian displacement for the 241 crystal structures, including 16 disordered structures, is only 0.095 Å (0.084 Å for the 225 ordered structures). R.m.s. Cartesian displacements above 0.25 Å either indicate incorrect experimental crystal structures or reveal interesting structural features such as exceptionally large temperature effects, incorrectly modelled disorder or symmetry breaking H atoms. After validation, the method is applied to nine examples that are known to be ambiguous or subtly incorrect.

## Abstract 2

The accuracy of a dispersion-corrected density functional theory method is validated against 241 experimental organic crystal structures from *Acta Cryst.* Section E.

## 1. Introduction

In principle, theoretical calculations could supply independent data about molecular crystal structures to complement experimental data. This idea is certainly not new and there are ample examples in the literature (for examples using quantum-mechanical calculations to supplement X-ray powder diffraction data see *e.g.* Smrčok *et al.*, 2008 ▶; Ávila *et al.*, 2009 ▶; Florence *et al.*, 2009 ▶). There are several occasions where such independent information can be very useful:

(i) As a supplement to low-quality or medium-quality experimental data such as powder diffraction data, especially when preferred orientation is present. This is relevant for crystal structures for which high-quality experimental data cannot be obtained, as may be the case for metastable polymorphs, for crystals measured in a diamond–anvil cell, for crystal structures of highly insoluble compounds such as organic pigments or for co-crystals obtained through grinding. The calculations can provide additional data that might resolve questions about possible disorder or space-group ambiguities.
(ii) To decide if an unusual feature is truly novel or just a problem with the interpretation of the experimental data, where ‘novel’ by definition implies that the feature cannot be verified against the existing literature and therefore excludes the use of databases.
(iii) To determine the positions of H atoms.
(iv) To decide between two structural models in case the experimental data are ambiguous.
(v) As an automated routine check on the correctness of experimental crystal structures.

The fifth application may seem the most obvious one, but it is deliberately listed at the end as it is a rather negative and trivial application, whereas it is our aim to present a method that can be used in more constructive and ambitious ways. These five applications have much in common, and there are no clear delineations between them, so most of the examples that will be given in this paper could be thought of as belonging to more than one category.

For completeness, and to avoid confusion, we would like to mention explicitly that using energy calculations to complement powder diffraction data to *validate* crystal structures is fundamentally different from using energy calculations to *solve* crystal structures from powder diffraction data: the latter requires the generation of trial structures and evaluation of their energies, a task for which the calculations presented in this paper would be too slow by several orders of magnitude. The distinction becomes even more subtle when energy calculations are used to decide between multiple structural models, all of them the result of a previous solution step: in that case the final crystal structure can be said to have been *determined* by energy calculations, but the crystal structure was not *solved* by energy calculations.

Although computational methods are commonplace these days, calculations on molecular crystal structures as a complement to and independent validation of experimental organic crystal structures are still not routine. Owing to their large system size and low symmetry in comparison to inorganic crystal structures, all pure quantum-mechanical calculations that might be accurate enough are prohibitively slow. One particular class of *ab initio* methods, density functional theory (DFT) calculations, is nowadays applicable to crystal structures with unit-cell sizes of up to several thousand Å^3^ on hardware available at the price of a diffractometer. DFT calculations, however, do not incorporate long-range dispersive interactions (part of the van der Waals interactions) which are particularly important in molecular crystals. As a result, so far most theoretical calculations in crystallographic journals have been limited to calculations on isolated molecules, dimers or clusters (to keep the systems sizes small) or on ionic compounds (see *e.g.* Smrčok *et al.*, 2008 ▶), or required the experimental unit cell to be kept fixed to avoid the crystal from expanding due to a lack of dispersion forces (see *e.g.* Ávila *et al.*, 2009 ▶; Florence *et al.*, 2009 ▶). On those occasions where calculations on true molecular crystal structures have been performed, *e.g.* with force fields, these have suffered from rather large and generally unknown errors, which made it difficult to rely on theoretical calculations. This lack of reliability applies equally to the crystal *structure*, *i.e.* the unit-cell parameters and atomic coordinates, as well as to the crystal *energy*.

In order for theoretical calculations on molecular crystal structures to become useful, their error must be small and must be known. It is the ambitious aim of this paper to validate a computational method whose results, especially the lattice parameters and the atomic coordinates, are so accurate that their information content and reliability are on a par with medium quality experimental data. In 2005 Neumann & Perrin published a paper in which they combined the plane-wave DFT code *VASP* (Kresse & Furthmüller, 1996 ▶; Kresse & Hafner, 1993 ▶; Kresse & Joubert, 1999 ▶) with an in-house parameterized dispersion correction. The combination of plane-wave DFT with a dispersion correction solves all the problems associated with calculations on molecular crystal structures in a very elegant manner: the use of plane waves allows fully quantum-mechanical calculations on periodic systems in a very natural manner, whereas the addition of a dispersion correction yields lattice energies that are, at least in theory, reliable even for molecular crystal structures. The best validation of the accuracy of the *energies* from this dispersion-corrected DFT (d-DFT) method came in 2007, when it predicted all four crystal structures in the Crystal Structure Prediction Blind Test correctly (Day *et al.*, 2009 ▶). In the present paper we will validate the accuracy of the *structures* from this d-DFT method against a large set of high-quality experimental organic crystal structures. Once the accuracy of the d-DFT method has been established by demonstrating that it is able to reproduce a large validation set of high-quality experimental organic crystal structures, the method can be applied with confidence to problems for which experimental data are hard or impossible to obtain.

## 2. Methods

The d-DFT energy minimizations were carried out with the computer program *GRACE*, which uses the computer program *VASP* for single-point pure DFT calculations. *GRACE* implements an efficient minimization algorithm to reduce the number of expensive single-point DFT calculations, and *GRACE* augments the pure DFT energies with a dispersion correction from hybridization-dependent isotropic atom–atom potentials. The details are given in Neumann & Perrin (2005 ▶); we mention here only that we use the Perdew–Wang-91 functional and a plane-wave energy cut-off of 520 eV. The dispersion-correction parameters for iodine were kindly provided by Dr J. Kendrick of the Institute for Pharmaceutical Innovation in Bradford; the dispersion-correction parameters for boron and bromine came from in-house parameterizations.^1^ All dispersion-correction parameters were parameterized against low-temperature (2–130 K) crystal structures and the d-DFT method was intended to reproduce unit-cell parameters at essentially 0 K. No dispersion-correction parameters were available for charged atoms: the parameters of the corresponding neutral species were used. The convergence criteria for the minimization were < 0.003 Å for the maximum Cartesian displacement (including H atoms), < 2.93 kJ mol^−1^ Å^−1^ for the maximum force and < 0.00104 kJ mol^−1^ per atom for the energy difference between the last two minimization steps.

The energy optimizations were divided into two steps: first an energy optimization with the unit cell fixed, followed by a second step with the unit cell free, starting from the energy-minimized crystal structure from the first step
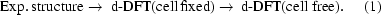
This two-step procedure has a computational advantage. From a numerical perspective, the energy of certain strong interactions such as chemical bonds is very sensitive to the atomic positions and small experimental uncertainties can result in large initial forces. At the beginning of the minimization procedure, when the optimization algorithm has no or only approximate information about the anisotropy of the curvature of the potential energy hypersurface, such forces can result in a large step in the wrong direction, ultimately leading to the structure getting trapped in a less favourable side minimum. The robustness of the minimization procedure is improved if ‘hard’ degrees of freedom, in practice intramolecular degrees of freedom, are minimized first. With respect to separating hard and soft degrees of freedom, the above scheme is not perfect since in the first minimization the soft molecular translations and rotations are adjusted together with the intramolecular degrees of freedom. In fact, in order to avoid getting trapped in a side minimum, for one crystal structure it turned out to be necessary to apply a three-step optimization procedure, with the unit-cell parameters, the molecular positions and the molecular orientations being held fixed for the first minimization
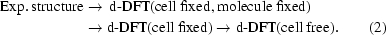
The three-step procedure requires more CPU time than the two-step procedure, and the three-step procedure should only be used if there are reasons to suspect that the crystal structure may have ended up in a side minimum.

Since pure DFT optimizations, without dispersion correction, are common in the crystallographic literature, almost invariably with the experimental unit cell kept fixed during the optimization, the calculations were repeated with pure DFT with the experimental unit cell kept fixed for comparison. The pure DFT calculations with fixed unit cell were carried out merely to reassure other authors that such calculations are indeed meaningful, and these calculation will only be mentioned briefly as part of the discussion.

Unless otherwise indicated, the experimental space-group symmetry was used, which imposes certain constraints on unit-cell parameters, atomic positions and *Z*.

For validation, two test sets will be used:

(i) a test set of crystal structures that can be assumed to be correct;
(ii) a test set of crystal structures that are known to be ambiguous or wrong.

For a test set of correct crystal structures, all 249 organic crystal structures from the August 2008 issue of *Acta Cryst.* Section E were downloaded, with permission. *Acta Cryst.* Section E is an open access journal, making the test set publicly available to all. Two crystal structures contained silicon and six contained phosphorus, two elements for which the dispersion correction has not yet been parameterized. These eight crystal structures had to be omitted from the test set, leaving 241 crystal structures. These 241 crystal structures cover a wide spectrum of molecular crystal structures including sugars, a high-energy material, drug molecules, chiral molecules, disordered structures, hydrates, solvates, salts and a range of space groups, functional groups and elements (C, H, B, Br, Cl, F, I, N, O and S). Three crystal structures are polymorphs of earlier determinations, but the test set contains no pairs of polymorphs. There were 16 disordered crystal structures which had to be adjusted manually before minimization. These disordered structures were not included in the validation set and will be discussed separately, leaving 225 crystal structures for the validation set.

For the test set of incorrect crystal structures, we took four structures that were known to be wrong. Two were from the literature (examples 1 and 6) and two turned up among the 225 structures in the *Acta Cryst.* Section E test set (examples 8 and 9). Four more crystal structures were added as examples where structure solution from powder diffraction data had yielded ambiguous results (examples 3, 4, 5 and 7). These structures require individual discussion and they are described below in §4.

Each crystal structure was energy-optimized in two ways: with the experimental unit-cell parameters kept fixed and with the unit cell allowed to vary. This provides us with a set of 225 times three crystal structures: the experimental crystal structure plus the two optimized structures. By comparing any two out of those three crystal structures and calculating the volume difference, the energy difference, the r.m.s. or the maximum Cartesian displacement with or without H atoms *etc.*, a large number of possible quality measures can be calculated. Moreover, two quality measures can be plotted against each other to generate two-dimensional scatterplots, quadratically increasing the number of plots. Several one-dimensional quality measures were explored in some detail, but one turned out to be the most relevant one for the purpose of discriminating between correct and incorrect crystal structures: the r.m.s. Cartesian displacement between the experimental crystal structure and the fully optimized crystal structure (including unit cell), excluding H atoms.

‘Cartesian displacement’ is not uniquely defined when the unit cells of the two crystal structures to be compared are different, as is the case when we compare the experimental crystal structure to the d-DFT optimized structure with the unit cell allowed to vary. In this work the Cartesian displacement for an atom in two crystal structures (1) and (2) is
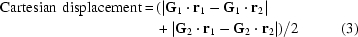
where **r**_*i*_ are the fractional coordinates of the atoms in crystal structure *i*, and **G**_*i*_ is the transformation matrix from fractional to Cartesian coordinates for crystal structure *i*. This definition of Cartesian displacement has the advantages that it is symmetric with respect to the two structures to be compared, that it varies smoothly upon smooth distortions of either or both of the two structures to be compared, and that there is no need for a user-defined parameter such as the number of molecules used for the comparison.

## 3. Results and discussion

### 3.1. Unit-cell volumes

**Figure 1:**
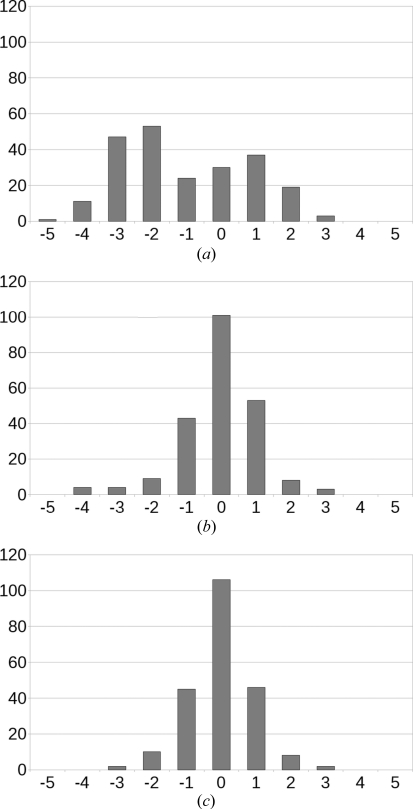
Distribution of volume discrepancies [in %, calculated as 100% × (*V*_d-DFT_ − *V*_Exp_)/*V*_Exp_] for the 225 *Acta Cryst.* Section E crystal structures after energy optimization with the d-DFT method. (*a*) Raw data; (*b*) when taking into account a fitted linear temperature correction; (*c*) when omitting six outliers (see text).

The reproduction of unit-cell volumes has already been described in the Neumann & Perrin (2005 ▶) paper and does not discriminate between correct and incorrect structures; we mention here that for the 225 crystal structures in the test set, the root mean square deviation (r.m.s.d.) in the unit-cell volume with the d-DFT method was 2.1%. The influence of the temperature at which the experimental structure had been determined was assessed by fitting a simple linear correction model, *V*_expected_(*T*_Exp_) = *V*_d-DFT_(*T*_d-DFT_) · (1 + *k* · (*T*_Exp_ − *T*_d-DFT_)). *V*_expected_ is the expected unit-cell volume, *T*_Exp_ is the temperature at which the crystal structure was measured, *V*_d-DFT_ is the unit-cell volume after energy-minimization, *T*_d-DFT_ is the apparent temperature of the d-DFT method and *k* is a linear expansion coefficient; *k* and *T*_d-DFT_ are the parameters that were fitted. According to our simple linear correction model, the d-DFT method produces unit-cell volumes that on average correspond to *T*_d-DFT_ = 150 K, whereas the average thermal expansion for the 225 organic crystal structures was *k* = 0.00016 K^−1^. The r.m.s.d. in the unit-cell volume was reduced to 1.1% after including the linear correction, and Fig. 1 ▶ shows a clear sharpening of the distribution. The odd asymmetry in the histogram in Fig. 1 ▶(*b*) is entirely due to six outliers (ci2620, ci2632, cv2431, hb2751, hb2754 and hb2762) that were all reportedly measured at 123 K, five by the same author and one from an author in geographical proximity, but that were more likely measured at room temperature as judged from their atomic displacement parameters (ADPs); without these six outliers, there are no discrepancies greater than ±3% and after fitting *T*_d-DFT_ and *k* again, the distribution is symmetric (Fig. 1 ▶ *c*). Expressed as averages instead of r.m.s.d.s, the average discrepancy in the unit-cell volume with the d-DFT method was −1.1%, in good agreement with the −1.0% from the original d-DFT paper;^2^ the reader is reminded that the dispersion-correction parameters were parameterized against low-temperature crystal structures, and a small contraction of experimental unit cells upon energy minimization is therefore expected. Without dispersion correction, *i.e.* when using pure DFT, the average volume discrepancy is about +20%.

### 3.2. R.m.s. Cartesian displacements

**Figure 2:**
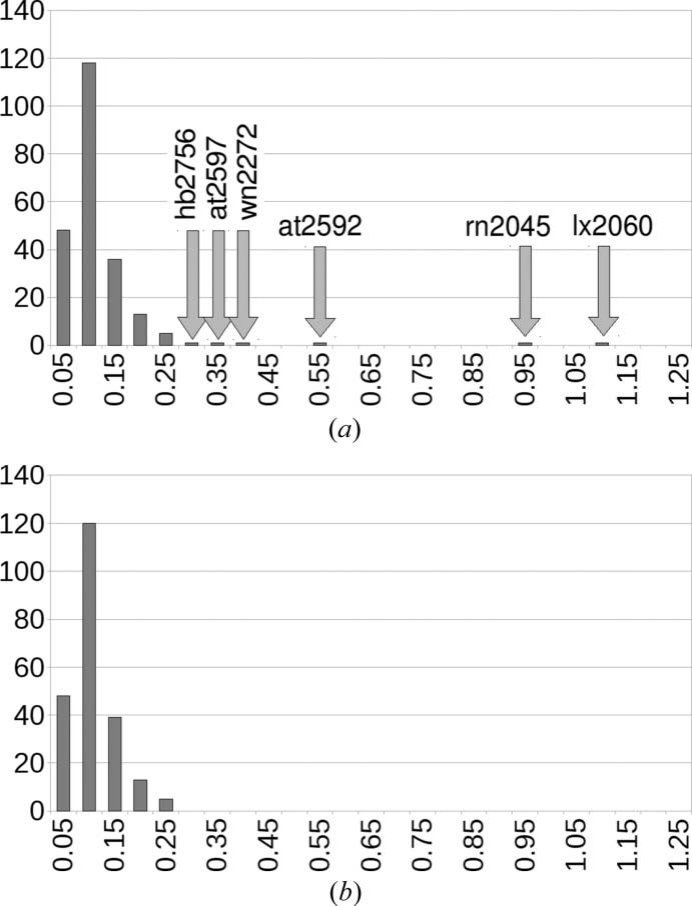
Distribution of the r.m.s. Cartesian displacements excluding H atoms of the experimental crystal structures *versus* the d-DFT(cell free) structures. (*a*) Initial results, (*b*) after analysing and correcting the three outliers and the three structures in the tail of the distribution (see text). The *x* axis labels indicate the upper limit of the range of each bin (Å).

Fig. 2 ▶ shows the distribution of the r.m.s. Cartesian displacements of the experimental crystal structures *versus* the d-DFT(cell-free) structures. H atoms are omitted throughout all comparisons in this paper, if only to eliminate the effect of reorientating methyl groups upon energy minimization.

As can be seen from the graph in Fig. 2 ▶(*a*), the initial energy minimizations showed three clear outliers, which are the crystal structures at2592 (Guo *et al.*, 2008 ▶), rn2045 (Choi *et al.*, 2008 ▶) and lx2060 (Xu & Hu, 2008 ▶).

Visual inspection of the crystal structure of lx2060 immediately revealed a missing hydrogen in the experimental crystal structure. *checkCIF* had issued a level G alert for the hydrogen-deprived C atom. Our findings were brought to the authors’ attention and the crystal structure was re-refined and published as an erratum (Xu & Hu, 2010 ▶). The r.m.s. Cartesian displacement fell from 1.07 to 0.13 Å.

rn2045 actually optimized to another minimum, which is generally caused by large forces at the start of the energy minimization. Controlled energy optimization in three stages as described above reproduced the experimental crystal structure of rn2045 without any problem, and the r.m.s. Cartesian displacement dropped from 0.91 to 0.10 Å. r2045 is a room-temperature structure.

The large r.m.s. Cartesian displacement of at2592 could not be explained: the crystal structure appeared to be correct and applying the three-stage energy minimization did not make a difference. The large average displacement was solely due to a 0.5 Å translation of the molecule as a whole along the **b** axis; the unit cell, molecular geometry, molecular orientation and molecular position along the **a** and **c** axes were virtually identical in the experimental and the energy-minimized structures. Even subtle asymmetry in the molecular geometry in spite of the symmetrical molecular topology was reproduced exactly. This shift in the **b** direction was also observed when the unit cell was kept fixed and when the space-group symmetry was lowered from *Pbca*, *Z*′ = 1 to *P*1, *Z*′ = 8. It was noticed, though, that at2592 was a room-temperature structure, and Dr A. D. Bond of the University of Southern Denmark offered to redetermine the at2592 crystal structure at 100 K. We are pleased to report that at 100 K the experimental structure corresponds virtually exactly to the energy-minimized structure (Bond *et al.*, 2010 ▶), and the r.m.s. Cartesian displacement decreases from 0.51 to 0.10 Å. In other words, this shift turns out to be an exceptionally large temperature effect.

The three worst outliers in Fig. 2 ▶ can hence be removed. For lx2060 and rn2045 this implies a correction to the experimental structure and to the computational method, respectively. For at2592, both the experimental structure and the calculated structure were correct within their respective domains of application.

Having been able to explain the worst three outliers, it is still interesting to look at the three structures that form the tail of the distribution in the left-hand side of Fig. 2 ▶. These are the three crystal structures hb2756 (Li *et al.*, 2008 ▶), at2597 (Chu *et al.*, 2008 ▶) and wn2272 (Luo *et al.*, 2008 ▶), with r.m.s. Cartesian displacements of 0.30, 0.34 and 0.40 Å (for reference, the maximum in Fig. 2 ▶ is at an average r.m.s. Cartesian displacement of 0.075 Å).

In hb2756 the two *n*-butyl chains are clearly disordered, which is obvious both from the large ADPs and from the small *sp* ^3^–*sp* ^3^ C—C distances, which range from 1.405 to 1.487 Å. It is clear that our static energy minimization is not able to reproduce this dynamic effect. As hb2756 is disordered, it should not be included in our test set.

In at2597 the slightly larger r.m.s. Cartesian displacement turned out to be due to incorrectly placed H atoms. Manual correction of the H atoms followed by energy minimization caused the r.m.s. Cartesian displacement to drop from 0.34 to 0.09 Å. This crystal structure provides a good example of how d-DFT can be used to determine positions for H atoms, and will be described in more detail below (see §4.8).

In wn2272 the slight distortion of the crystal structure upon energy minimization again turned out to be due to incorrectly modelled H atoms in the experimental structure. In this case the H atoms should have been modelled as disordered, as described in §4.9. When corrected the r.m.s. Cartesian displacement is only 0.11 instead of 0.40 Å.

We conclude that the slightly larger r.m.s. Cartesian displacements in hb2756, at2597 and wn2272 can all be explained and made to vanish if we adhere to our principle that disordered structures should not be included in the test set and that errors in structures should be corrected before they are included. The right-hand side of Fig. 2 ▶ shows the final distribution of the r.m.s. Cartesian displacements for the 225 crystal structures. The 225 energy-minimized crystal structures, with the unit cell free, have been deposited.^3^

The *Acta Cryst.* Section E test set contains 27 crystal structures of molecular salts, whereas the dispersion-correction parameters were parameterized against compounds without formal charges; the distributions of the quality measures show that these crystal structures (*i.e.* atomic coordinates and unit-cell parameters) are reproduced as accurately as those of neutral molecules. (This does not necessarily mean, however, that the *energies* of the crystal structures of these molecular salts are of the same accuracy as those of neutral molecules.)

The r.m.s. Cartesian displacement excluding H atoms upon energy minimization with the unit cell free is an appropriate measure for the correctness of an experimental crystal structure. For the 225 single-crystal structures from *Acta Cryst.* Section E, the r.m.s. Cartesian displacement correctly and unambiguously identified three outliers.

There is one structure that could be considered a false positive: the structure of at2592 at room temperature cannot be reproduced with the d-DFT method. Redetermination of the experimental crystal structure at 100 K provides unambiguous proof that the d-DFT method and the experimental method correspond to the same minimum, although such an amount of additional experimental effort is never desirable and may not always be possible. A computational solution is possibly the use of a Molecular Dynamics (MD) or Monte Carlo (MC) simulation. An MD or MC simulation requires a force field, ideally one of an accuracy comparable to the d-DFT calculations. This can be achieved by parameterizing a dedicated force field for the compound under consideration against reference data calculated with the d-DFT method. The details of how such a tailor-made force field can be parameterized are described elsewhere (Neumann, 2008 ▶). Out of the 225 crystal structures in the test set, 118 were determined at room temperature (defined here as between 290 and 300 K); this means that based on the examples used in this paper, less than 1% of molecular crystal structures display a large temperature effect. d-DFT calculations (but not pure DFT calculations, see below) can be used to screen a database of molecular crystal structures for those with possible interesting temperature effects.

### 3.3. Timings

**Figure 3:**
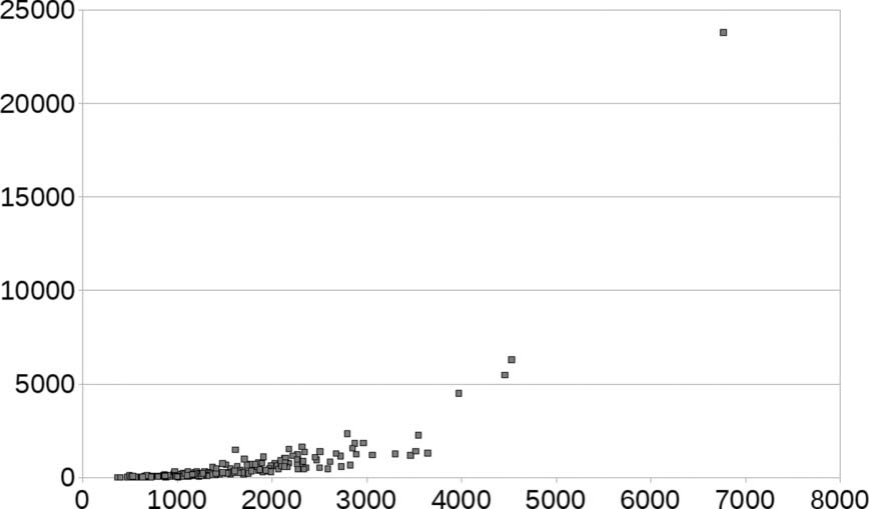
Duration (h) of the energy minimizations as a function of reduced unit-cell volume (Å^3^) normalized to one core.

Fig. 3 ▶ shows the timings of the energy minimizations as a function of reduced unit-cell volume. The energy-minimizations were parallelized over multiple cores, the exact number of cores depending on the reduced unit-cell volume, but the timings have been normalized to reflect how long the energy minimization would have taken on a single core. The calculations took about one month on our full cluster, which consists of 64 1-GHz 64-bit quad-core Opteron processors.

### 3.4. Disordered structures

16 out of the 241 structures are disordered. The disorder can be grouped into three categories.

(I) Crystal structures in which part of a molecule can have two distinct conformations, each of which can be energy-minimized separately and remains a stable minimum. The disorder in these structures is static and can be modelled with our static calculations. An example is a disordered —CF_3_ group. This sort of disorder is present in bt2740, ci2628, ci2633, fb2101, lh2658, lh2661, tk2283, xu2430 and xu2435.
(II) Crystal structures containing at least one atom with two possible positions that are very close to each other, and which both converge to the same position when energy-minimized. It appears that the disorder in these structures is purely a dynamic effect, which cannot be modelled with static energy minimizations. Three structures in our test set exhibit this kind of disorder: bx2164, gk2158 and hb2758. hb2756, which was mentioned above as containing disorder, should probably also be considered as type (II) disordered, although the authors modelled the disorder through large isotropic atomic displacement parameters rather than through multiple atomic positions with fractional occupancies.
(III) Crystal structures in which some of the H atoms need to be ‘symmetry-disordered’: the positions of a few H atoms are not commensurate with the space-group symmetry of the non-H atoms. Such structures can only be energy-minimized in a subgroup of the experimental space group. wn2272 (experimental space group *C*222_1_, *Z*′ = 1, subgroup *P*2_1_2_1_2_1_, *Z*′ = 2), discussed elaborately below, is an example of such a case (although we included it in the test set as ‘not disordered’, because it was published as ordered). The two other examples are bi2287 (experimental space group *C*2/*c*, *Z*′ = 1, subgroup *Cc*, *Z*′ = 2) and cs2083 (experimental space group *Pbcm*, *Z*′ = ½, subgroup *Pbc*2_1_, *Z*′ = 1). bh2169 contains type (II) disorder combined with a different kind of disorder not belonging to types (I), (II) or (III): the structure contains a methanol molecule with an occupancy of 25%.

**Figure 4:**
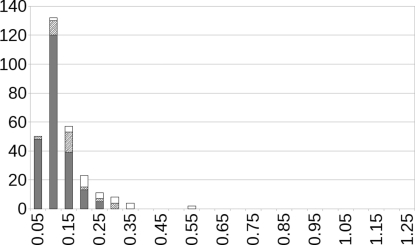
R.m.s. Cartesian displacements, without H atoms, upon energy-minimization for the disordered structures with the unit cell free. In dark grey, the 225 crystal structures from the reference test set. Hatched bars correspond to the lower of the two values; white bars to the higher value (see text). The hatched and the white bars have been multiplied by a factor of two for clarity. Scales for the *x* and *y* axes as for Fig. 2 ▶.

An in-depth discussion of the 16 disordered crystal structures is beyond the scope of this paper, and only a few interesting features will be described in brief. The crystal structures with type (II) or type (III) disorder are trivial, because all models of structures with type (II) disorder converge to the same structure and structures with type (III) disorder merely require a space-group reduction. The r.m.s. Cartesian displacement upon energy-minimization is below 0.15 Å for all type (III) structures and below 0.25 Å for all type (II) structures (see Fig. 4 ▶), with the exception of hb2756 as discussed above. When cs2083 is minimized in subgroup *Pbc*2_1_, the molecule tilts slightly (2.0°) out of the 001 plane and the disorder in the methyl group cannot be reproduced; additional calculations in other space groups would be necessary to fully understand the nature of the disorder, but these were outside the scope of this paper.

A subtle issue for type (I) disordered crystal structures needs mentioning: the two distinct minima generally correspond to slightly different unit cells. Given that crystallographers use a single set of unit-cell parameters for their measurements and refinements, it is an interesting question how the magnitude of the difference between these two unit cells affects the crystallographic figures of merit of disordered crystals. In a real crystal the local unit cell may be partially imposed by the surrounding unit cells and may be similar for the two alternative structures if the disorder occurs at random (*i.e.* does not form domains). Minimizing the two minima each in their own unit cell provides the two systems with more degrees of freedom than justified. The energies of the two minima are therefore slightly inconsistent, making it difficult to compare them. Both conformers of bt2740, fb2101, lh2658, lh2661 and tk2283 are reproduced very well, with r.m.s. Cartesian displacements smaller than 0.15 Å and negligible energy differences. For ci2628, ci2633 and xu2430, one of the two conformers is reproduced much better than the other (r.m.s. Cartesian displacements around 0.10 Å for one conformer, around 0.30 Å for the other), and the energy differences between the two conformers are starting to become significant. The conformer with 15% occupancy in xu2430 is especially unlikely according to the d-DFT calculations with an r.m.s. Cartesian displacement of 0.52 Å. In xu2435 the energy difference between the two conformers is virtually zero, but both distort by more than 0.20 Å.

For type (I) disordered structures, two experimental alternatives correspond to two energy-minimized structures; for type (II) structures, two experimental alternatives correspond to one energy-minimized structure. In both cases two r.m.s. Cartesian displacement values must be calculated, which have been included separately in Fig. 4 ▶ as a minimum and a maximum value. Fig. 4 ▶ clearly shows that for disordered crystal structures at least one experimental alternative is reproduced very well by the d-DFT calculations, and in most cases the accuracy of the d-DFT calculations for both alternatives of a disordered structure is only slightly lower than for ordered structures. Based on the small sample of 16 structures available here, we conclude that the d-DFT calculations can be applied to disordered crystal structures with only a small loss of accuracy.

### 3.5. Database of energy-minimized crystal structures

The unprecedented high accuracy of the d-DFT method in reproducing crystal structures of molecular compounds, including their unit-cell parameters, can be used to create a database of energy-minimized crystal structures. There are several advantages a collection of energy-minimized experimental crystal structures might have over a collection of experimental crystal structures.

(i) First, when a crystal structure is flagged up as ‘incorrect’, *e.g.* by *checkCIF*, and a possible cause is identified, it is currently in many cases virtually impossible to prove that the manually corrected structure is indeed the correct structure without access to experimental data such as the original structure factors. In such cases the d-DFT method is now able to act as a reliable and independent referee, without the need for additional experiments. This means that suspicious crystal structures do not need to be merely discarded, but can be actively corrected and included in a database of energy-minimized crystal structures, even if experimental data can no longer be obtained.
(ii) Another advantage is that after energy-minimization, crystal structures determined from powder diffraction data and those determined from single-crystal data are of the same accuracy.
(iii) Last but not least, in a database based on energy-minimized experimental crystal structures, the coordinates of the H atoms are as reliable as the coordinates of the non-H atoms.

### 3.6. Pure DFT calculations

**Figure 5:**
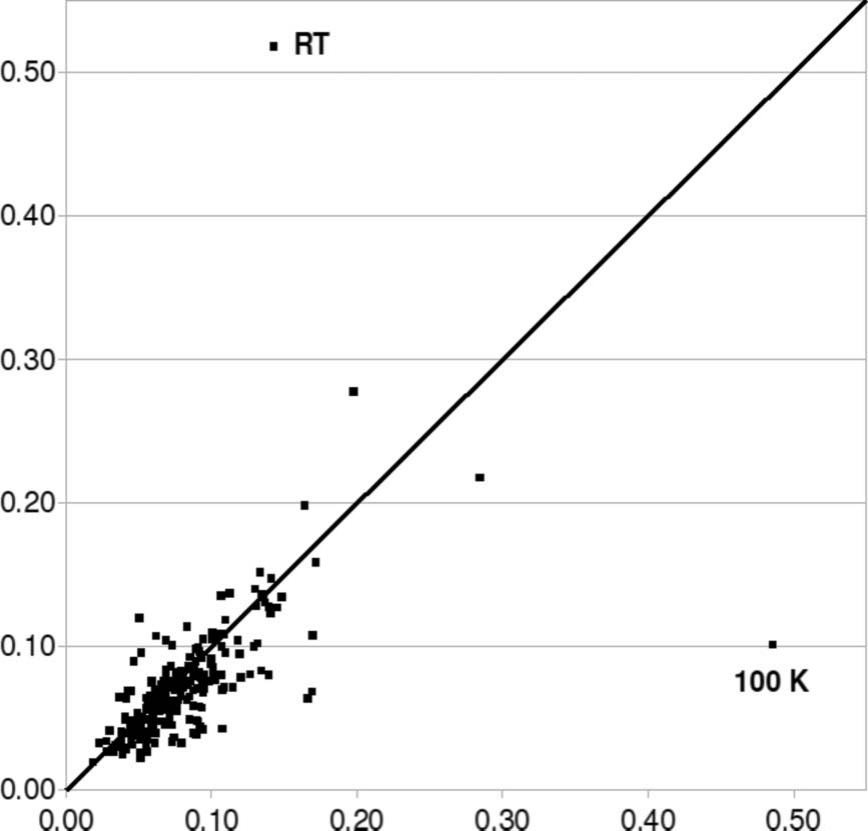
R.m.s. Cartesian displacement (Å), excluding H atoms, upon energy minimization with a fixed experimental unit cell with pure DFT (*x* axis) *versus* dispersion-corrected DFT (*y* axis). The line *y* = *x* is drawn to guide the eye. The two outliers are at2592 at 100 K and at room temperature.

For pure DFT calculations (without dispersion correction) the experimental unit cell must be imposed, greatly restricting the number of possible quality measures: essentially only the r.m.s. Cartesian displacement can be used. Fig. 5 ▶ shows the r.m.s. Cartesian displacement upon minimization with pure DFT *versus* the r.m.s. Cartesian displacement upon minimization with dispersion-corrected DFT; the experimental unit cell was kept fixed for both. Structure at2592, the structure with the significant temperature effect, was included twice, at 100 and 298 K (see below). Although there is a considerable range, it is clear that the overall distributions for both methods are very similar. The main message of Fig. 5 ▶ is therefore that if the experimental unit cell is kept fixed, pure DFT and dispersion-corrected DFT perform equally well for molecular crystal structures.

Three minor remarks can be made about Fig. 5 ▶. First, the structure at2592 is clearly an outlier, but whether the pure DFT or the d-DFT method reproduces the experimental structure more accurately is temperature dependent. The room-temperature structure is reproduced very well by pure DFT, whereas for d-DFT the agreement with the structure at 100 K is excellent. Which of these two at2592 structures should be included in Fig. 5 ▶? On the one hand, the room-temperature structure seems the fairer choice: that is the structure that was published, and it is the structure that corresponds to the physical conditions that matter for real-life applications. There is one strong argument though: why is the 100 K structure more relevant in the context of this paper? The dispersion-correction part of the d-DFT method was parameterized against low-temperature crystal structures with the explicit aim of devising a method that would be able to reproduce organic crystal structures at 0 K as accurately as possible and that was considered a first step only; the influence of temperature was considered to be an independent problem, to be solved at a later date as a second step. By selecting the 100 K structure, this separation between static, 0 K, energy minimizations and the influence of temperature as two independent problems is preserved. Both structures are included in Fig. 5 ▶, and we leave it up to the reader to decide which structure to consider the more relevant one.

Second, although pure DFT and d-DFT perform equally well when the experimental unit cell is kept fixed, this does not change the fact that we observed that the distribution of the r.m.s. Cartesian displacements is sharper and gives a clearer divide between correct and incorrect structures if the unit cell is also optimized; without dispersion correction, the experimental unit cell must be imposed.^4^

Third, from comparison against the *y* = *x* line, the structures minimized with d-DFT appear to have systematically lower r.m.s. Cartesian displacements. Indeed, for 147 of the 226 structures (65%) the r.m.s. Cartesian displacement of the structure minimized with d-DFT is lower than that of the structure minimized with pure DFT. In other words, even when the experimental unit cell is kept fixed dispersion-corrected DFT performs marginally better than pure DFT for about two thirds of all organic crystal structures.

However, these minor remarks should not distract from the main message in Fig. 5 ▶: provided that the experimental unit cell is kept fixed, DFT with and without dispersion correction perform essentially equally well and for most purposes either can be used to validate organic crystal structures.

## 4. Example cases

To start with the most trivial case, we begin by demonstrating the use of the d-DFT method for detecting incorrect experimental crystal structures. As mentioned, the kind of applications in the d-DFT method listed in §1 overlap to a certain extent and the assignments of example cases to individual categories are not cast in stone.

### 4.1. Example 1: Rietveld refinement of a wrong crystal structure

Buchsbaum & Schmidt (2007 ▶) published a Rietveld refinement of a crystal structure which they knew to be incorrect: they fitted the crystal structure of the β polymorph of quinacridone to the experimental X-ray diffraction powder pattern of the γ polymorph. In spite of being wrong, the Rietveld refinement passed a check list of seven items and the authors posed the question how one could have known that the crystal structure was incorrect. The paper was highlighted in the IUCr Newsletter (2007, Volume 15, Number 4).

**Figure 6:**
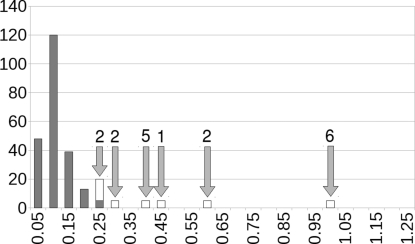
R.m.s. Cartesian displacements, without H atoms, upon energy-minimization with the unit cell free for the example cases. In dark grey, the 225 crystal structures from the reference test set. White bars correspond to crystal structures that are known to be incorrect; the white bars have been multiplied by a factor of two for clarity. The numbers above the arrows refer to the respective examples. Scales for the *x* and *y* axes as for Fig. 2 ▶.

This is a trivial case for the d-DFT method. The non-planar molecular geometry in the incorrect ‘experimental’ crystal structure is such an unrealistic geometry for the aromatic ring system that with an r.m.s. Cartesian displacement of 0.45 Å the structure clearly stands out as an outlier (Figs. 6 ▶ and 7 ▶). With only 0.06 Å the correct crystal structure offers a far more realistic alternative.

### 4.2. Example 2: Editorial on article retractions

While this paper was being written, an editorial appeared in *Acta Cryst*. Section E announcing the retraction of 70 crystal structures from the journal because of scientific fraud (Harrison *et al.*, 2010 ▶). The fraud consisted of taking a single set of experimental intensity data to publish multiple papers, with the authors changing one or more atoms from the original, genuine, crystal structure to produce what appeared to be genuine structure determinations of new compounds. As far as is known, these ‘derived’ crystal structures do not correspond to real crystal structures and should be considered incorrect. It is therefore an interesting question if the d-DFT method would have uncovered these crystal structures as suspicious.

45 of the crystal structures are inorganic or organometallic and were not considered. 20 of the remaining crystal structures are organic, but contain multiple hydrogen-bond donors and acceptors that often do not form chemically sensible hydrogen bonds in the crystal structures as published. With r.m.s. Cartesian displacements of the order of 0.50 Å, the d-DFT calculations clearly indicate that these structures in their published form are incorrect, but the authors could simply have rebutted that this is caused only by erroneously placed H atoms (similar to wn2272 and at2597 below) and that working through all permutations of possible hydrogen-bonded networks would eventually lead to a plausible crystal structure. Manually adjusting hydrogen-bonded networks followed by multiple minimizations represents a very substantial amount of work, of the order of at least one week for each of the 20 structures, an amount of work that could not be justified given that it is already known that the underlying ‘plausible structure’ does not exist. For one of these structures, hk2325, the r.m.s. Cartesian displacement is only 0.20 Å, and this is the only incorrect crystal structure that our criterion is not able to identify as incorrect, or at least as suspicious.

This leaves five crystal structures that at first glance appear credible (at2444, hk2347, hk2357, hk2367 and hk2389) and these five structures were energy-minimized with the d-DFT method. Disappointingly, the results are not as clear cut as one might have hoped (Fig. 6 ▶). The five crystal structures, though known to be questionable, are fairly sensible. Although none of the five structures yields figures of merit that would qualify it as a ‘good’ crystal structure, only one of them (hk2389) distorts enough upon energy-minimization that it could have been confidently rejected as incorrect (r.m.s. Cartesian displacement of 0.56 Å). The other four structures produce r.m.s. Cartesian displacements that are all just within or just beyond the limits of what would have been acceptable for a good crystal structure, and could all be argued to be structures that are correct and that happen to yield r.m.s. Cartesian displacements that lie in the tail of the distribution. This is hard to refute, especially since these crystal structures are room-temperature structures, and minor discrepancies can easily be blamed on the d-DFT calculations.

The 20 crystal structures with many alternative hydrogen-bonded networks highlight a more general problem if the d-DFT method is to be used to confirm the correctness of crystal structures: the burden of proof should be on the person that determined the crystal structure, not on the person that wants to use the structure. That, however, is only a fair expectation if the d-DFT method is available to the entire academic scientific community in a manner that allows energy-minimizations to be fast and affordable.

### 4.3. Example 3: *Pn*2_1_ *a* or *Pnma*?

Since certain symmetry operators cause extinctions whereas others do not, generally speaking multiple space groups share the same extinction conditions. In such cases a crystal structure can be solved and refined in the space group with the lowest number of symmetry operators, and the decision as to which space group to assign to the final structure must be based on the atomic coordinates. If only powder diffraction data are available, the final atomic coordinates after Rietveld refinement in the subgroup may not be reliable enough to decide on the final space group. Rietveld refinements in both space groups will probably result in very similar figures of merit, and it is difficult to decide if slightly better figures of merit for the subgroup are significant, and not merely caused by the increased number of degrees of freedom (due to a decrease in the number of symmetry operators). This was the case, for example, for a 1:1 caffeine:acetic acid co-crystal that could be obtained exclusively by grinding, and therefore only powder diffraction data were available (Trask *et al.*, 2005 ▶). The systematic absences pointed to *Pnma* or its subgroup *Pn*2_1_ *a* as the space group, and both molecules in the structure have internal mirror symmetry and are thus capable of occupying a position on a mirror plane. After structure solution in the subgroup *Pn*2_1_ *a*, the larger caffeine molecule was situated exactly on what would have been a mirror plane in *Pnma*, but the smaller acetic acid molecule was slightly tilted out of that plane. Rigid-body Rietveld refinement in both space groups gave slightly better figures of merit for the subgroup, as expected, but the difference was judged to be insignificant, especially considering that the slightly better figures of merit were achieved with no less than twice as many degrees of freedom. Combined with the observation that in the 1:2 co-crystal, which could be solved from single-crystal data, both molecules also occupy mirror planes (space group *C*2/*m*), the space group *Pnma* could be assigned with a high degree of confidence.

With the d-DFT method, the space-group assignment could have been checked without any reference to further experimental data. Conclusive proof of the true space group would require full characterization of the free-energy hypersurface at room temperature, to establish if the molecular orientations in *Pnma* correspond to a true minimum, but our calculations are currently restricted to a single energy-minimization on the lattice-energy hypersurface at 0 K. Optimizing the crystal structure twice, starting from the Rietveld refinements in *Pn*2_1_ *a* and in *Pnma*, shows that the two models converge to essentially the same structure, with comparable lattice energies. After energy minimization, the acetic acid molecule in the *Pn*2_1_ *a* structure lies on the virtual mirror plane, just like the caffeine molecule. This is a strong indication that the published space-group assignment *Pnma* was correct.

### 4.4. Example 4: Decide on possible disorder from powder diffraction data

Detecting disorder in a molecular crystal structure if only powder diffraction data are available is complicated by the fact that the disorder divides the few electrons per C, N, O or F atom over multiple positions, blurring the structural features even further. Individual isotropic atomic displacement parameters, let alone individual anisotropic atomic displacement parameters, can seldom be meaningfully refined from powder diffraction data due to increased peak overlap, reduced peak intensities and strong correlation with the background at high 2θ angles.

The crystal structure of bt2740 from the *Acta Cryst.* Section E test set contains a disordered —CF_3_ group, and the disorder is reproduced extremely well by our calculations in every respect: after energy optimization, the two alternative structures have nearly identical unit cells, nearly identical energies, and the positions of the F atoms in the two geometries of the —CF_3_ group correspond very closely to those found in the experimental structure.

This stands us in good stead for tackling the possible case of a disordered —CF_3_ group in a crystal structure from powder diffraction data. Pigment Yellow 154 (PY 154) is an organic pigment containing a —CF_3_ group. Like all pigments, it is virtually insoluble in most solvents, preventing the growth of single crystals from solution. The crystal structure was therefore solved from laboratory powder diffraction data by van de Streek *et al.* (2009 ▶). As —CF_3_ groups are prone to disorder, the Rietveld refinement was carried out with and without a disorder model for the —CF_3_ group. Unfortunately, owing to the limited information content in laboratory powder diffraction data, the two Rietveld refinements showed no significant differences. Applying Ockham’s razor, the authors decided to publish the crystal structure without disorder. We can now use the d-DFT method to check for the presence of disorder by energy-optimizing the crystal structure twice, starting with both orientations of the —CF_3_ group, to establish if both orientations correspond to a stable minimum. Upon energy-minimization with the d-DFT method, both disorder models converge to the same structure, with the same energy. In other words, there is only one stable minimum for the orientation of the —CF_3_ group, and although it might be dynamically disordered (rotating essentially freely), the disorder cannot be described as the presence of two distinct minima. The stable minimum corresponds to the published crystal structure, with an r.m.s. Cartesian displacement of only 0.07 Å.

### 4.5. Example 5: O=C—NH_2_ ambiguity

**Figure 8:**
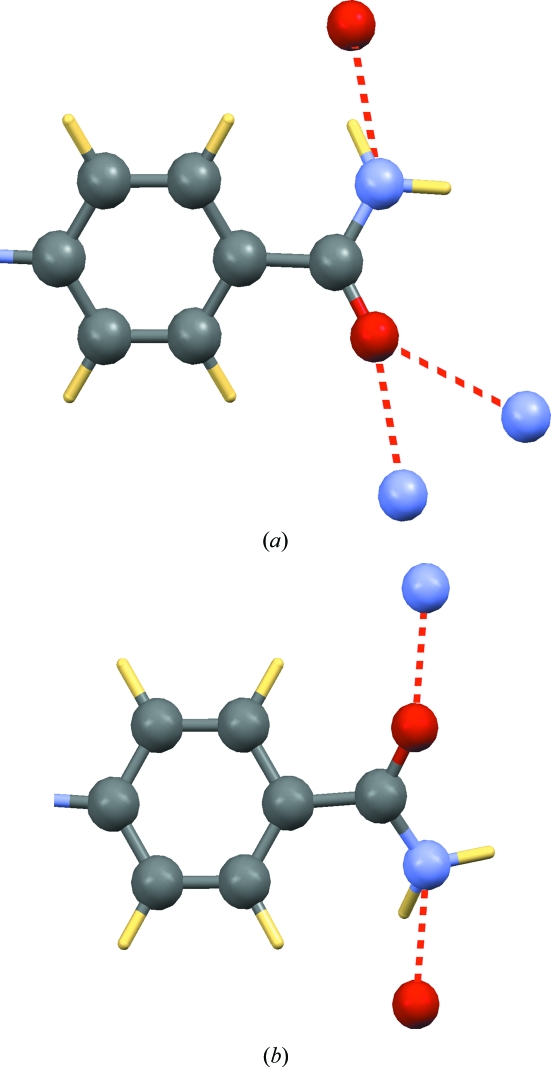
The two alternative orientations of the terminal amide group in the crystal structure of PY 181, each forming an infinite chain of hydrogen bonds with itself. Hydrogen bonds are shown as red dashed lines. The two alternatives are indistinguishable from powder diffraction data.

An amide group being planar, a rotation over 180° exchanges the O and the N atom. The one electron difference in electron density between an O atom and a N atom renders the two atoms indistinguishable when only laboratory X-ray powder diffraction data are available. In the crystal structure of Pigment Yellow 181 (PY 181), which was determined from laboratory X-ray powder diffraction data (Pidcock *et al.*, 2007 ▶), the amide group forms an infinite hydrogen-bonded chain with itself: a rotation of the amide group over 180° therefore keeps the infinite hydrogen-bonded chain intact (Fig. 8 ▶). In this case it is difficult to select the correct model with confidence from the experimental data alone. In the original paper, force field methods were used to decide on the correct orientation of the O=C—NH_2_ group; here we present the results from d-DFT calculations for the two possible models.

The energy difference between the two alternatives is 23 kJ mol^−1^ in favour of the published structure (the left-hand side in Fig. 8 ▶). In the four successful crystal structure predictions mentioned in §1 (Day *et al.*, 2009 ▶), the relative energies computed with the d-DFT method successfully reproduced energy differences of the order of 1 kJ mol^−1^, proving beyond reasonable doubt that the correct orientation was published.

This example differs from the other examples in two ways. First, energies are compared rather than Cartesian displacements. Second, in this example we already know that one of the two models is wrong and there is an alternative available, which makes it trivial to establish which model is the more plausible alternative. A more relevant question in the context of the present paper is: given only the wrong crystal structure, can we detect it as such? With an r.m.s. Cartesian displacement of 0.35 Å, the incorrect structure clearly falls outside the range expected for correct crystal structures (Fig. 6 ▶). The correct alternative shows an r.m.s. Cartesian displacement of only 0.09 Å.

### 4.6. Example 6: A novel heterocyclic compound

In 2008 Fang and co-workers (Fang *et al.*, 2008 ▶) published an erratum for their 2007 paper (Fang *et al.*, 2007 ▶) describing a ‘novel’ heterocyclic compound. It turned out that several elements had been misassigned, and the ‘novel’ heterocyclic compound (sum formula Na_2_C_4_H_18_N_2_O_15_) was, in fact, common borax (sum formula Na_2_B_4_H_20_O_17_; Levy & Lisensky, 1978 ▶). This compound contains sodium, an element for which the dispersion correction has not been parameterized. Each Na atom is, however, octahedrally coordinated by six O atoms, which shield the sodium from the rest of the structure. It is to be expected that pure DFT is able to describe the Na—O bonds, and that the rest of the structure will be held together by ionic interactions and the dispersion-correction contribution from the non-Na atoms. For each Na atom, the unit cell contains ten non-Na, non-H atoms and ten H atoms.

**Figure 9:**
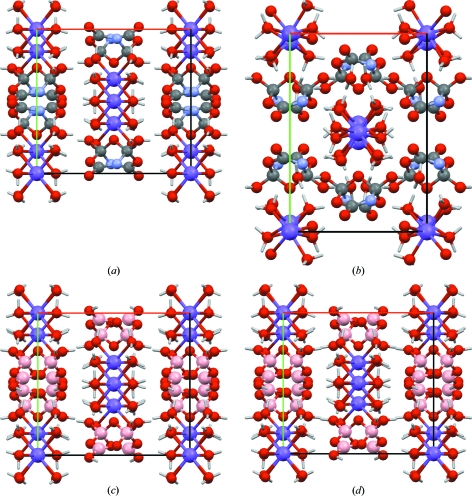
A ‘novel’ heterocyclic ring. (*a*) and (*b*) the crystal structure of a ‘novel’ heterocyclic ring with misassigned elements. The published crystal structure is shown on the left, the minimized structure is shown on the right. (*c*) and (*d*) the crystal structure of common borax. The experimental crystal structure is shown on the left, the minimized structure (including optimization of the unit cell) is shown on the right. The incorrect element assignments in the structure in the top row clearly do not correspond to a stable minimum according to the d-DFT method, whereas the correct structure is reproduced perfectly. Na and B atoms are shown in purple and in pink, respectively.

Upon energy-minimization the correct crystal structure hardly changes, with an r.m.s. Cartesian displacement of 0.08 Å. The crystal structure with the incorrect element assignments rearranges substantially, the r.m.s. Cartesian displacement being 0.99 Å (Fig. 9 ▶).

### 4.7. Example 7: A non-planar commercial organic pigment

van de Streek and co-workers published the crystal structures of six commercially produced organic pigments, Pigment Orange (PO) 36, PO 62, Pigment Yellow (PY) 151, PY 154, PY 181 and PY 194, determined from laboratory X-ray powder diffraction data (van de Streek *et al.*, 2009 ▶). In the crystal structures of PO 36, PO 62, PY 151, PY 154 and PY 181, the angle between the phenyl ring and the benzimidazolone moiety is 1.45, 1.69, 4.53, 1.20 and 8.50°, indicating that the conjugated π-systems are planar, as expected for the commercial phases of organic pigments. For PY 194, however, this angle is 18.56°. For PY 194, experimental data to a real-space resolution of only 2.6 Å were available. Furthermore, it can be argued that the electron density of a planar molecule would lead to a peak of high intensity in the powder pattern: if therefore, conversely, the intensity of this peak were to be affected by preferred orientation, the (incorrect) lower peak intensity would correspond to less electron density being present in that plane, and some of the atoms must then be forced out of that plane during the Rietveld refinement. The two aromatic ring systems each being restrained to be planar, an obvious degree of freedom available to the refinement for pushing atoms out of that plane would be the angle between the two aromatic systems. It would therefore be justified for a suspicious reader to wonder if the slightly unusual molecular geometry in the crystal structure of PY 194 is perhaps due to preferred orientation and is not slightly unusual, but merely slightly wrong.^5^ This is an excellent case for the d-DFT method to prove its usefulness: as stated in the paper by van de Streek *et al.*, no crystal structures of similar molecules had been published before, and the five crystal structures of similar molecules in the same paper, if anything, indicate that the molecular geometry of PY 194 is suspicious. All six crystal structures from the paper were therefore energy-optimized with the d-DFT(cell-free) method. All six crystal structures were reproduced very well (all six r.m.s. Cartesian displacements smaller than 0.15 Å), and in the energy-optimized crystal structure of PY 194 the angle between the phenyl ring and the benzimidazolone ring is 20.16°; for PO 36, PO 62, PY 151, PY 154 and PY 181 the corresponding angles in the energy-optimized crystal structures are 4.45, 4.54, 3.38, 4.66 and 8.83°. We can now be confident, without the need for additional experiments or even access to a sample of the compound, that the crystal structure as determined from low-resolution laboratory powder diffraction data is correct.

### 4.8. Example 8: An intramolecular N—H⋯S hydrogen bond

The determination of the positions of H atoms in crystal structures determined from X-ray diffraction data is probably the most obvious application of computational methods, because of the intrinsic problems in locating H atoms experimentally owing to their low X-ray scattering power. For glycerol, where the OH hydrogen atoms had not been determined experimentally, coordinates were proposed based on calculations (Mooij *et al.*, 2000 ▶) and for β-d-allose the experimental coordinates of the OH hydrogen atoms were questioned and a new set of coordinates was proposed, also based on calculations (van Eijck *et al.*, 2001 ▶). The *Acta Cryst.* Section E test set contains two nice examples of incorrect hydrogen positions.

**Figure 10:**
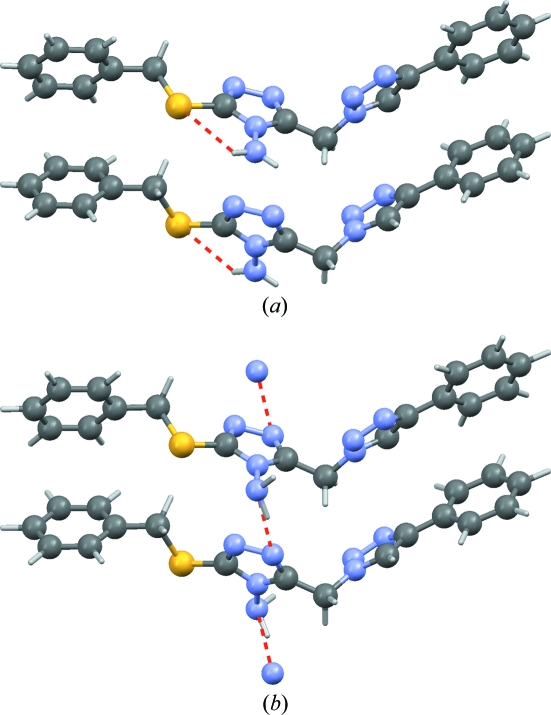
The coordinates of the —NH_2_ hydrogen atoms in at2597 according to the authors of the experimental crystal structure (*a*) and according to the d-DFT method (*b*): the N—NH_2_ group is not planar, there is no intramolecular N—H⋯S hydrogen bond present in the structure and an intermolecular N—H⋯N hydrogen bond was missed. Hydrogen bonds are shown as dashed red lines.

at2597 (Chu *et al.*, 2008 ▶) has already been singled out above because it was one of the three structures in the tail of the r.m.s. Cartesian displacement histogram (Fig. 2 ▶) with a value of 0.34 Å. Visual inspection of the crystal structure before and after energy-minimization showed a substantial rearrangement of the hydrogen-bonding pattern: according to the authors, the N—NH_2_ group is planar and forms ‘an intramole­cular N—H⋯S hydrogen bond’, whereas according to the d-DFT method the N—NH_2_ group is tetrahedral and forms an intermolecular N—H⋯N hydrogen bond (Fig. 10 ▶). However, this still did not explain the slightly larger r.m.s. Cartesian displacement, as the r.m.s. Cartesian displacements in Fig. 2 ▶ were calculated without taking H atoms into account. The substantial rearrangement of the hydrogen-bonding pattern suggested that the positions of the H atoms in the starting structure were far away from their equilibrium positions, and this in turn suggested that the structure may have minimized to the wrong minimum. This was checked by manually changing the orientation of the two —NH_2_ hydrogen atoms; subsequent energy-minimization confirmed that the d-DFT structure now reproduced the non-H atoms in the experimental structure very well with an r.m.s. Cartesian displacement of only 0.09 Å. Fig. 10 ▶ shows the hydrogen-bonding pattern as published (left) and the hydrogen bonding pattern as arrived at through d-DFT calculations (right). Surprisingly, the energies of our two calculated minima, differing in the orientation of the two H atoms of the —NH_2_ group, are equal within the accuracy of our method. This means that it is highly likely that the protons are not static in the structure, but are dynamically delocalized. This might explain why the authors were able to write ‘all H atoms were located in difference Fourier maps’: there may have been multiple weak minima, two of which happened to correspond to what the authors considered to be reasonable positions for the —NH_2_ hydrogen atoms. Nonetheless, the authors should still have checked their own assumptions more closely before refining the H atoms in a restrained geometry: the N—NH_2_ group is certainly not planar, and in none of our alternative models is an intramolecular N—H⋯S hydrogen bond present.

### 4.9. Example 9: Disordered H atoms

**Figure 11:**
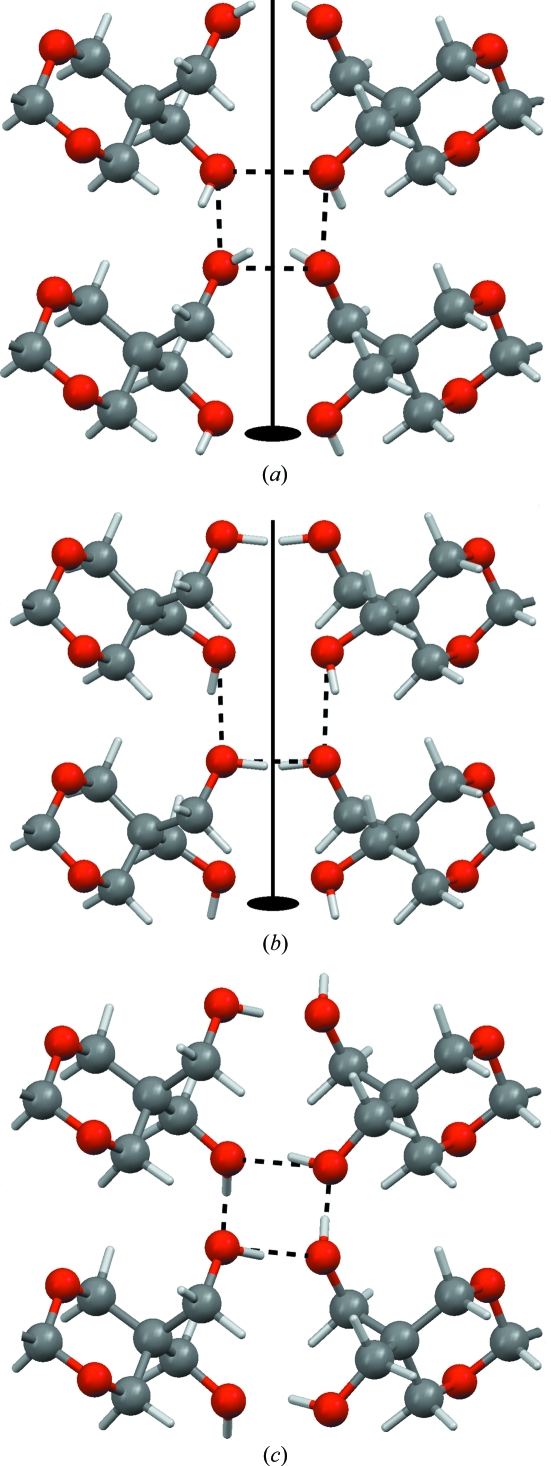
The hydrogen-bonded helix in the crystal structure of wn2272; the hydrogen bonds of a single helix are drawn as black dashed lines. (*a*) Experimental structure, (*b*) minimized in the experimental space group *C*222_1_, *Z*′ = 1 and (*c*) minimized in *P*2_1_2_1_2_1_, *Z*′ = 2. In the experimental space group, a twofold axis prevents the O—H hydrogen atoms from forming reasonable hydrogen-bond geometries, whereas in *P*2_1_2_1_2_1_, *Z*′ = 2 perfect helices can be formed.

wn2272 (Luo *et al.*, 2008 ▶) has been mentioned before because it is a crystal structure from the tail of the r.m.s. Cartesian displacement distribution (Fig. 2 ▶). In wn2272 a combination of disorder and incorrect hydrogen positions plays a role: disordered H atoms. Owing to the low X-ray scattering power of H atoms, the overall crystal symmetry is determined by the non-H atoms and the H atoms are only added after the structure has been solved. In the case of wn2272 the true positions of the H atoms are not commensurate with the space-group symmetry of the non-H atoms: the O—H groups are expected to form infinite helices of hydrogen bonds, but in the published structure the twofold rotation axes at 0,*y*,¼ running midway between two O—H groups prevent the formation of hydrogen bonds with sensible O—H⋯O geometries. Energy optimization with the experimental space group imposed is equally unsuccessful in producing sensible hydrogen bonds, and for the same reason. If the space-group symmetry is lowered from *C*222_1_, *Z*′ = 1 to *P*1, *Z*′ = 8, there are eight possible combinations of directions for the four helices in the unit cell. Models were prepared for the four more symmetrical combinations, and the models were energy-minimized. After energy-minimization, the new space-group symmetry was determined. For the model with all pairs of neighbouring helices running in opposite directions, so as to minimize the dipole moment throughout the crystal structure, the space-group symmetry is *P*2_1_2_1_2_1_, *Z*′ = 2 after energy-minimization (within 0.025 Å); *P*2_1_2_1_2_1_ is a maximal subgroup of *C*222_1_. If the —OH groups and all H atoms are ignored, the space-group symmetry is the experimental space group *C*222_1_, *Z*′ = 1 (within 0.05 Å). This model also corresponds to the lowest energy, albeit by a negligibly small margin. The energy differences between the various permutations of directions of helices are small (less than 0.5 kJ mol^−1^; *RT* = 2.47  kJ mol^−1^ at room temperature), and in the true crystal structure the directions of the helices are probably at least to a certain extent arbitrarily distributed. Per helix, the hydroxyl groups are ordered, but when averaged over the entire crystal the hydroxyl groups are disordered, presumably exactly 50/50. The energy of the structure energy-minimized in *P*2_1_2_1_2_1_, *Z*′ = 2, is 41 kJ mol^−1^ more favourable than when energy-minimized in the experimental space group (unit-cell parameters free in both cases, but α, β and γ forced to be 90° in both space groups). The r.m.s. Cartesian displacement upon energy-minimization, which is 0.40 Å when the experimental space-group symmetry is imposed, drops to 0.11 Å for *P*2_1_2_1_2_1_, *Z*′ = 2. Fig. 11 ▶ shows the experimental structure, the energy-optimized structure with the experimental space-group symmetry imposed and the structure as energy-optimized in *P*2_1_2_1_2_1_, *Z*′ = 2.

Unfortunately, the authors of wn2272 did not realise that the overall space-group symmetry had to be replaced by a more local view in order for the hydrogen bonds to make sense, as they claim (presumably in response to a level B alert from *checkCIF*): ‘For one of the two hydroxyl groups (O3), its hydrogen atom does not form a hydrogen bond to an adjacent acceptor atom. Other possibilities for placing hydrogen atoms on the two groups led to unacceptably short H⋯H interactions of less than 2 Å.’ The fact is that in the true crystal structure, both hydroxyl groups participate equally in the formation of infinite chains of excellent, cooperative hydrogen bonds without direct H⋯H interactions. Describing these hydrogen bonds correctly in the experimental space group *C*222_1_ would have required the hydroxyl groups to be refined as disordered. At first glance, it may seem slightly perverse to refine a H atom, with its single electron, over two positions. In the case of wn2272, though, the combination of chemistry and crystallography dictates that this be so, and this is also what the authors of, for example, bi2287 (Masuda, 2008 ▶) and cs2083 (Liu *et al.*, 2008 ▶) did when facing a similar problem.

### 4.10. Locating H atoms: co-crystal *versus* salt

Although not applicable to any of the examples used in this paper, it is an interesting question: can the d-DFT method be used not only to make minor adjustments to the geometries of experimentally determined H atoms, but to locate the H atoms if no experimental coordinates for H atoms are available, for example to decide whether a crystal structure is a co-crystal or a salt? The answer is, in principle, no. The decision whether a crystal structure is a co-crystal or a salt requires the comparison of two models. In the absence of reliable experimental data, the most natural parameter to compare would seem to be the energies of the two models, *cf*. the O=C—NH_2_ ambiguity example above. However, the excellent accuracy of the d-DFT energies has only been validated for the relative energies of polymorphs. It is known that DFT energies are in general less accurate when chemical bonds are broken or formed: when comparing energies of polymorphs, all chemical bonds in all crystal structures are the same, and any inaccuracies in heats of formation are cancelled. Unfortunately, this is no longer true when two crystal structures consist of different chemical entities, as is the case for a co-crystal *versus* a salt. Until the accuracy of the d-DFT method has been validated separately for this type of calculation, the d-DFT method must be considered unsuitable for a direct comparison of the energies.

The d-DFT method can take us a considerable way towards deciding between a co-crystal or a salt if at least some experimental data are available: namely reliable coordinates of the non-H atoms. In that case, we can energy-minimize both models and calculate the r.m.s. Cartesian displacement for each of them: after energy-minimization, one of the two models will fit the experimental coordinates of the non-H atoms better than the other model. If the difference is significant – and the bulk of this paper is devoted to describing test sets of numbers that can be used to quantify ‘significant’ – the model that provides the better fit can be confidently chosen as the one with the correct H-atom assignment. It could be argued that our discovery of the missing hydrogen in the structure of lx2060 (see above) is a trivial example of such an application. That such an approach can be successful for more subtle cases was demonstrated in a paper by Trask *et al.* (2005 ▶) on a caffeine-trifluoroactetic acid co-crystal. Severe disorder in the —CF_3_ groups of the *Z*′ = 2 crystal prevented the authors from locating the relevant H atoms in a Fourier difference map, even from single-crystal data. The protonation state of two caffeine molecules had to be established based on the geometry of their imidazole rings, which could be shown to be strongly dependent on the protonation state. Trask *et al.* used two database searches to obtain their two sets of reference values, but the principle is easily transferred to using two energy-minimizations with the d-DFT method.

However, in the case of lx2060 the missing H atom should have been trivial to spot, and no d-DFT minimization should have been necessary. In the caffeine-trifluoroactetic acid co-crystal the severe disorder would have made the d-DFT calculations less reliable. Also, in general, single-crystal data are good enough to locate the H atoms, whereas for powder diffraction data, where the d-DFT method might be useful, the coordinates of the non-H atoms may not be accurate enough to decide between the two models anyway. Therefore, the applicability of the d-DFT method to locate H atoms is limited, and in principle the d-DFT method cannot currently be used to decide between a co-crystal or a salt.

## 5. Conclusion

Dispersion-corrected DFT (d-DFT) is able to reproduce experimental organic crystal structures very accurately. Owing to this high accuracy it is possible to energy-optimize a proposed crystal structure and to use the discrepancies between the proposed and the energy-minimized crystal structure to decide about the correctness of the proposed crystal structure. The most useful quality measure is the r.m.s. Cartesian displacement excluding H atoms upon full energy-minimization (including unit-cell parameters). Exceptionally strong temperature effects can lead to confusion, but these are rare (< 1%) and only play a role in crystal structures determined at room temperature (∼ 50% of all organic crystal structures from *Acta Cryst.* Section E); molecular crystal structures determined at temperatures lower than 200 K are always reproduced very well. In turn, the d-DFT method provides us with a tool to screen a collection of room-temperature molecular crystal structures for temperature effects. Perhaps surprisingly, the d-DFT calculations appear to be reliable even for disordered crystal structures.

Pure DFT calculations can be very useful for also validating molecular crystal structures, but are limited to calculations with fixed unit cells.

Assigning correct hydrogen-bond geometries is easier and more reliable with d-DFT methods than with X-ray diffraction, and is cheaper, faster and easier with d-DFT than with neutron diffraction. *Locating* H atoms, however, *e.g.* to decide if a crystal structure is a salt or a co-crystal, requires further validation regarding the accuracy of the d-DFT energies involving breaking and forming bonds.

The high accuracy and reliability of the calculations allow the calculations to be used as a source of independent data that can be used to decide about subtle structural features in molecular crystal structures determined from low-quality experimental data, such as powder diffraction data. The d-DFT calculations can be used as a tool to decide on hydrogen-bond geometries, on the correct space group and on the presence of disorder.

## Supplementary Material

Supplementary material file. DOI: 10.1107/S0108768110031873/so5041sup1.txt

## Figures and Tables

**Figure 7:**
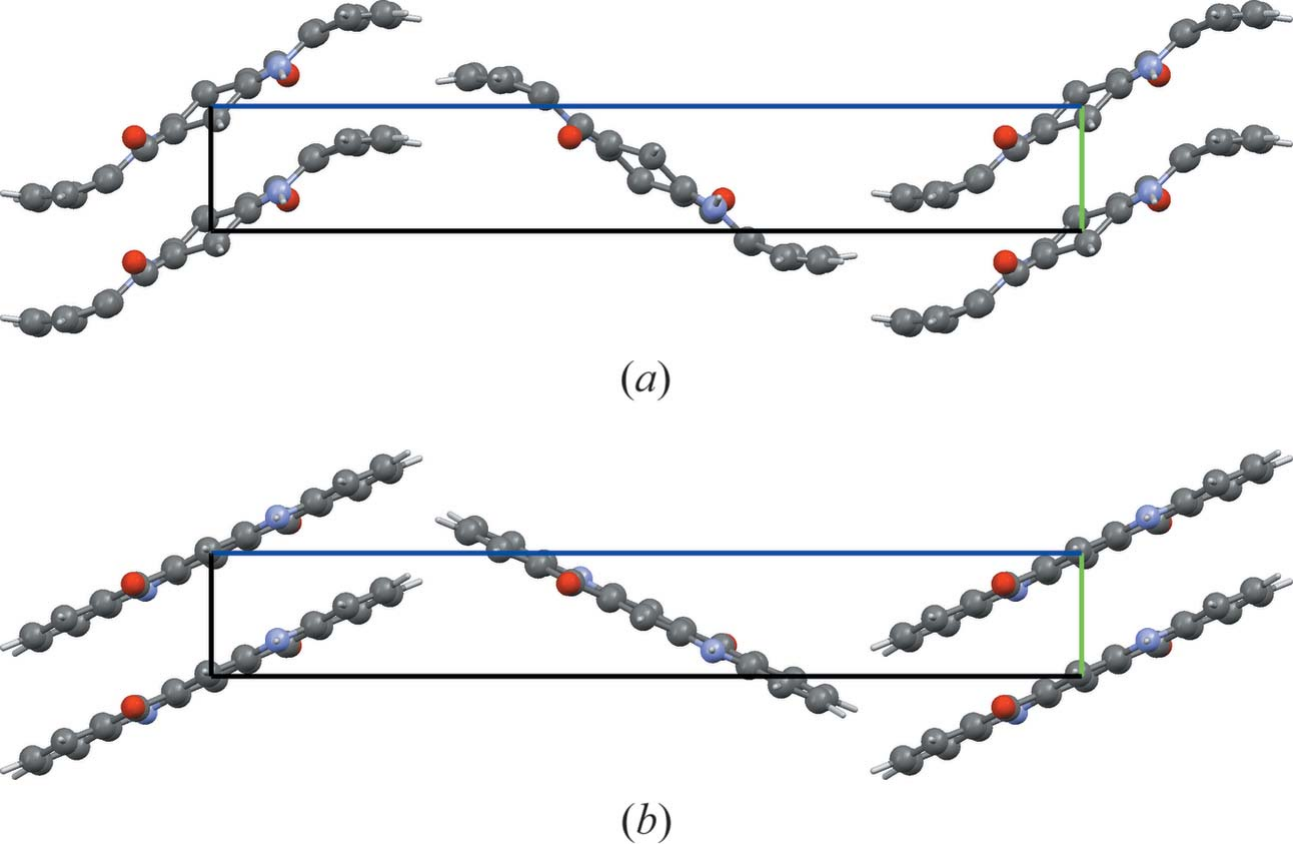
The incorrect crystal structure of the β polymorph of quinacridone. (*a*) The experimental crystal structure and (*b*) the energy-minimized structure.

## Footnotes

1 For bromine, *C*_6_ = 9776 kJ mol^−1^ Å^6^, crossover distance = 3.748 Å, for boron, *C*_6_ = 1934 kJ mol^−1^ Å^6^, crossover distance = 4.95 Å.

2 The average reduced to zero upon applying the fitted linear correction, but this is not a meaningful figure because the fitting procedure automatically makes the average discrepancy vanish.

3 Supplementary data for this paper are available from the IUCr electronic archives (Reference: SO5041). Services for accessing these data are described at the back of the journal.

4 This comparison is, however, partially unfair: in our work we focused on the histogram in Fig. 2 ▶ and scrutinized all six outliers until we were able to explain them. Considering how subtle some of the issues with the experimental crystal structures were, it is highly likely that more minor issues are still present in the test set, which were simply not discovered. It is possible that analysing more histograms and rectifying the remaining minor issues makes the other distributions slightly sharper as well.

5 In practice, information on the electron density is distributed much more evenly over the intensities of a powder diffraction pattern than we have assumed here, and it is unlikely that a crystal structure containing such an error could give such an excellent fit over the entire range of the pattern.
